# Examining subjective understandings of autistic burnout using Q methodology: A study protocol

**DOI:** 10.1371/journal.pone.0285578

**Published:** 2023-05-19

**Authors:** Jane Mantzalas, Amanda L. Richdale, Cheryl Dissanayake

**Affiliations:** Olga Tennison Autism Research Centre, La Trobe University, Bundoora, Victoria, Australia; The University of Sydney, AUSTRALIA

## Abstract

**Background:**

Early research indicates that autistic burnout is a chronic, debilitating condition experienced by many autistic people across the lifespan that can have severe consequences for their mental health, wellbeing, and quality of life. To date, studies have focused on the lived experiences of autistic adults, and findings suggest that a lack of support, understanding, and acceptance by others can contribute to the risk of autistic burnout. The study outlined in this protocol will investigate how autistic people with and without experience of autistic burnout, their families, friends, healthcare professionals and non-autistic people understand the construct of autistic burnout to identify commonalities and gaps in knowledge.

**Study and design:**

Q methodology will be used to investigate participants’ subjective understandings of autistic burnout. Q methodology is a mixed-methods design that is well-suited to exploratory research and can elucidate a holistic and comprehensive representation of multiple perspectives about a topic. Participants will complete a card sorting activity to rank how strongly they agree or disagree with a set of statements about autistic burnout and participate in a semi-structured interview to discuss their responses. A first-order factor analysis will be conducted for each participant group, followed by second-order factor analysis to compare the groups’ viewpoints. The interview data will provide additional insights into the factors.

**Conclusion:**

Q methodology has not previously been used to examine autistic and non-autistic people’s perspectives about autistic burnout. Projected study outcomes include enhanced understanding of the characteristics, risks, and protective factors of autistic burnout. The findings will have practical implications for improving detection of autistic burnout and identifying strategies to support autistic adults with prevention and recovery. The results may also inform the development of a screening protocol and identify potential avenues for future research.

## Introduction

‘Autistic burnout’ describes a state of debilitating physical, mental, and emotional exhaustion experienced by autistic people, resulting from demands associated with being autistic and living in a predominantly non-autistic world. These demands can include overwhelming sensory stimuli, social and communication differences, unexpected changes to routines, and a lack of reasonable accommodations [1–4]. Consequences of autistic burnout include reduced tolerance to sensory stimuli, difficulties with self-care and speech, social withdrawal, and more marked autistic characteristics. Autistic burnout can persist for months or even years, limiting participation in work, study, and social activities [1–4]. Moreover, it can affect autistic people across the lifespan, from childhood [5] to late adulthood, and severely impact their mental health, wellbeing, independence, and quality of life [1–4]. While the prevalence of autistic burnout remains unknown, it is estimated that there are approximately 78 million autistic people worldwide [6]; thus, even a conservative estimate of 1–2% prevalence of burnout suggests that over a million autistic individuals could be impacted. Myriad anecdotal reports imply this figure could be much higher.

Research indicates that poor autism awareness within social, healthcare, and family systems can influence the onset and recurrence of burnout through one or more of unreasonable expectations, misdiagnosis, and insufficient support for autistic people [2,4]. Autistic adults are more likely to experience co-occurring mental health conditions than adults in the general population [7], but often describe negative experiences within healthcare systems and a deep mistrust of healthcare providers [8–10]. Reasons include insufficient autism training and knowledge, negative attitudes about autism, previous misdiagnosis, the lack of available experts, and unwillingness to provide flexible care to accommodate the sensory and communication needs of autistic people [8–10]. Despite the global prevalence of autism (1:100 children) [6,11], healthcare providers acknowledge they receive little, if any, formal autism training which contributes to self-reported low confidence and competence treating autistic patients [12,13]. It is possible that healthcare professionals who lack experience working with autistic people are also unaware of autistic burnout.

Poor societal and community awareness can contribute to autistic burnout if autism is not recognised early, or the needs of autistic people are dismissed or ignored [1–4]. A pervasive lack of autism understanding and acceptance in society also underly negative stereotypes and stigma, and contribute to the high rates of bullying, sexual, and interpersonal victimisation experienced by autistic people [14]. Stigma from close relatives, spouses, or friends can become internalised and prevent autistic people from disclosing their autism diagnosis, limiting necessary accommodations and contributing to the risk of autistic burnout [1–4,10].

To avoid negative consequences associated with stigma, autistic people often ‘mask’ their autistic characteristics to ‘pass’ as non-autistic to achieve social inclusion and gain access to employment and education opportunities [15]. However, the prolonged use of masking strategies (e.g., preparing scripts before social interactions, faking eye contact, suppressing self-regulating behaviours) is cognitively exhausting and has been identified as a prominent risk factor for autistic burnout [1,2,4,15,16].Other risk factors for autistic burnout include poor self-awareness due to alexithymia and impaired interoception [1,2], stressful life events and transitions [1,2,4], co-occurring conditions (especially depression) [2,4], sensory sensitivities, and gender[1,3,4]. Autistic burnout can precede an autism diagnosis, especially in adulthood, as autistic people are often unaware of autistic burnout until after they have experienced it [1,2], highlighting the importance of understanding gaps in knowledge and improving awareness both within and outside the autistic community.

The study outlined here will be the first to examine autistic burnout from the perspective of autistic adults, non-autistic adults, healthcare providers, and family or friends of autistic adults. Two broad theoretical frameworks underpin this research. We adopt a social constructionist approach which assumes that the way individuals understand the world is the product of historical and cultural contexts, power relations and social interactions between people [17]. We assume that stakeholders’ perspectives about autistic burnout are influenced by how they view autism–specifically via the medical model or the social-relational model of disability. The former takes a deficit-based view of disabilities as something to be cured or fixed [18] while the social-relational model suggests that, while medical conditions may confer some inherent impairments, these become *disabling* if social factors (e.g., discrimination or unequal access to opportunities and resources) limit a person’s opportunity to participate in society [19].

The goals of the proposed research are to:

1. Understand the onset and recurrence of autistic burnout and prevention and recovery from the perspectives of these different groups of people.
2. Develop a holistic characterisation of autistic burnout to inform training and awareness initiatives and to improve the care, support, and mental health and wellbeing outcomes for autistic people.

Q methodology (or ‘Q’) was chosen to address the study goals because its abductive approach prioritises ‘making discoveries rather than testing our reasoning’ [20, p151]. Thus, no *a priori* assumptions or pre-determined hypotheses are offered [21]. Instead, theories will be proposed in hindsight, or *a posteriori*, to explain the emergent factors and understand their meaning from the perspective of the participants [22]. These theories can be tested experimentally in future research. Q methodology is well-suited to exploratory research and has previously been used to investigate the perspectives of patients and healthcare professionals regarding medical conditions such as irritable bowel syndrome and chronic back pain [23,24]. This approach has previously been used to understand how to best support autistic people in employment and higher education [25,26].

The presumption that opinions about autistic burnout are finite and can be analysed systematically to identify clusters of perspectives underlies this research [21,27]. Q studies holistically explore the dimensions of a topic, and their findings represent a snapshot of the participants’ subjective viewpoints [27]. The proposed study aims to capture *what* members of the four participant groups think about autistic burnout, not *how many* people agree or disagree [28]. In other words, this research is less concerned with ‘*who* said what about [autistic burnout]?’ than ‘*what* is currently being *said* about [autistic burnout]?’ [21, p86]. People’s viewpoints can change over time; therefore, results from a Q study only describe the perspectives of the individuals who take part. Rather than generalising the findings to other populations, generalisations tend to focus on the theories constructed to interpret and understand the emergent factors [22].

### Research questions

The research will seek to answer the following questions:

1. How do autistic adults understand autistic burnout?
2. How do parents and significant others of autistic adults understand autistic burnout?
3. How do healthcare professionals understand autistic burnout?
4. How do non-autistic adults understand autistic burnout?

## Methods

### Ethics

This study has received ethical approval from the La Trobe University Human Research Ethics Committee (reference number HEC21008).

### Study design

Q is a mixed-methods technique for studying human subjectivity that involves compiling a set of statements (Q-set) that represent the breadth of perspectives (not facts) about an issue and asking participants to rank and assign meaning to them (see Table 1 for glossary). Q differs from traditional R methodology studies (Table 1) in that the Q-set represents the sample and the participants (P-set) represent the variables. The goal is to understand the range of viewpoints participants hold rather than making inferences about the individuals themselves or generalising their responses to a wider population [22,27,28].

**Table 1:** Glossary of terms used in Q methodological research.

Term	Definition^a^
R methodology	Research methods that classify traits or tests as variables and participants as the sample, whose results are generalised to a broader population. Employs *by-variable* factor analysis.
Q methodology	A research method that classifies participants as variables and traits or tests as the sample, whose findings are not generalisable. Employs *by- person* factor analysis.
Concourse	A ‘universe’ of statements that comprises the spectrum of opinions about a topic or issue.
Q-set	A subset of statements from the concourse that represents the diverse range of viewpoints about a topic.
P-set	The participants in a Q study.
Q-sort	An activity that requires participants to subjectively rank the items in the Q set according to their level of agreement.
Q-grid Factor array	The matrix used in the Q-sort activity that resembles an inverted normal distribution. A composite Q-sort that exemplifies the viewpoint of a factor.

^a^ Source [22].

P-set selection is *theoretical* and underpinned by the assumption that there are finite opinions about autistic burnout [22,27,28]. Unlike R methodologies where larger samples are often required to achieve valid and reliable results, Q studies do not require many participants and power analysis is not used to calculate sample size [27]. An optimal sample in a Q study contains individuals who represent diverse perspectives relevant to the study’s goals [28]. Generally, a smaller P-set than Q-set is usually recommended (typically 40–60 participants), or one participant per two Q-set items [22].

Q data analysis involves correlating the participants’ opinions and analysing them using by-person (‘inverted’) factor analysis, where the resulting factors represent similar viewpoints among participants [20]. To date, research about autistic burnout has focused on the experiences of autistic people [1,2,4,5], but the proposed study will include individuals who may be affected by or affect, people’s experiences of autistic burnout. Through the Q-set, the participants’ perspectives about the aetiology, symptoms, and impact of autistic burnout and prevention and recovery strategies will be explored.

### Participants

#### Inclusion criteria

**Fig 1:**
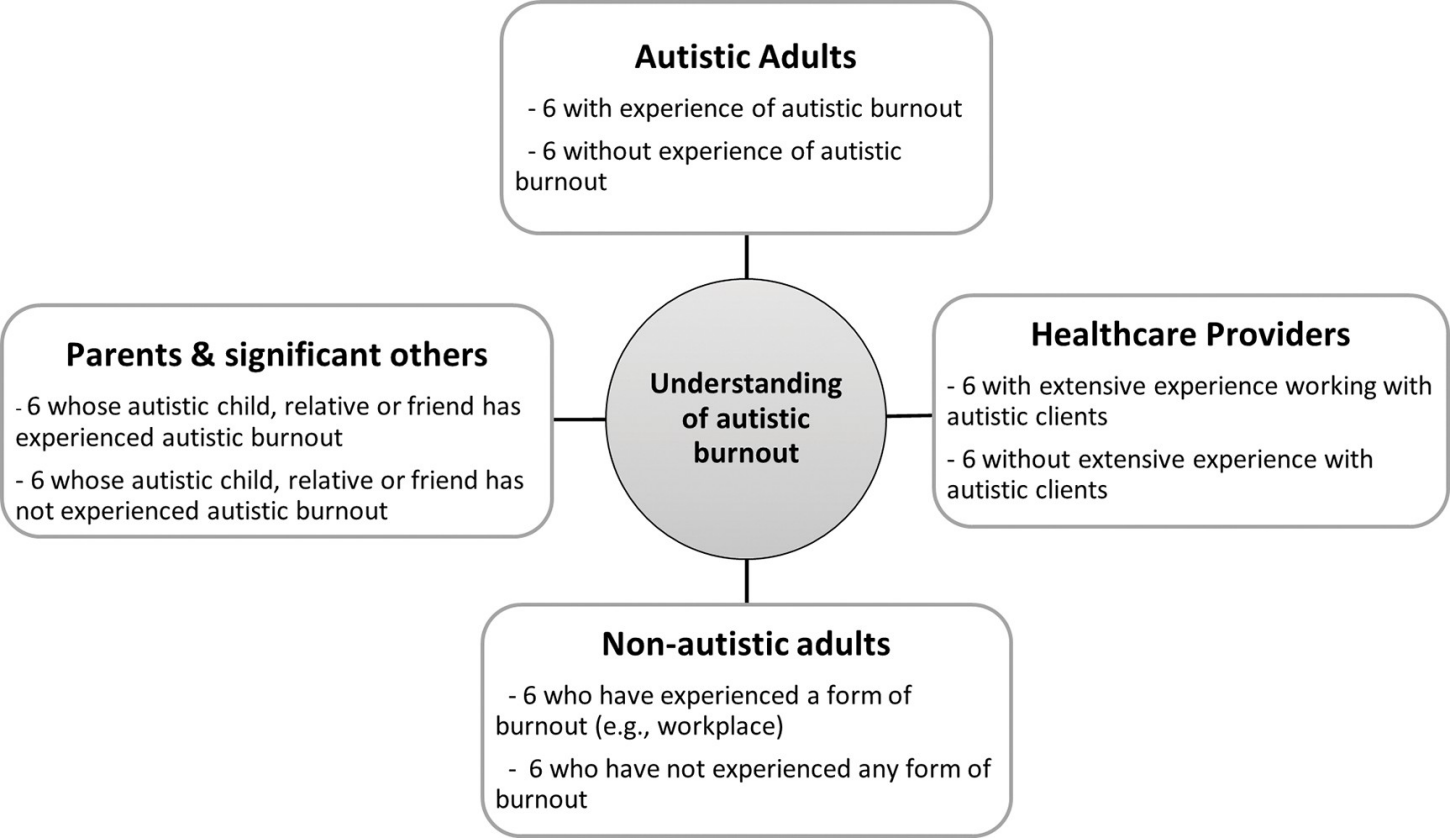
Diagram showing the structure of the four participant groups.

The study will be open to participants worldwide. Four participant groups will be recruited, each comprising 12 individuals (Fig 1). Eligible participants must be at least 18 years of age, able to read and communicate in English and have access to the internet. Participants in the autistic adult group must have a formal autism diagnosis. The healthcare provider group will include professionals who may work with autistic clients including psychologists, general practitioners, occupational therapists, and psychiatrists. The parents and significant others group will include parents, partners, spouses, siblings, close friends, or other relatives of an autistic adult (18+ years).

#### Structure of the four participant groups

**Fig 2:**
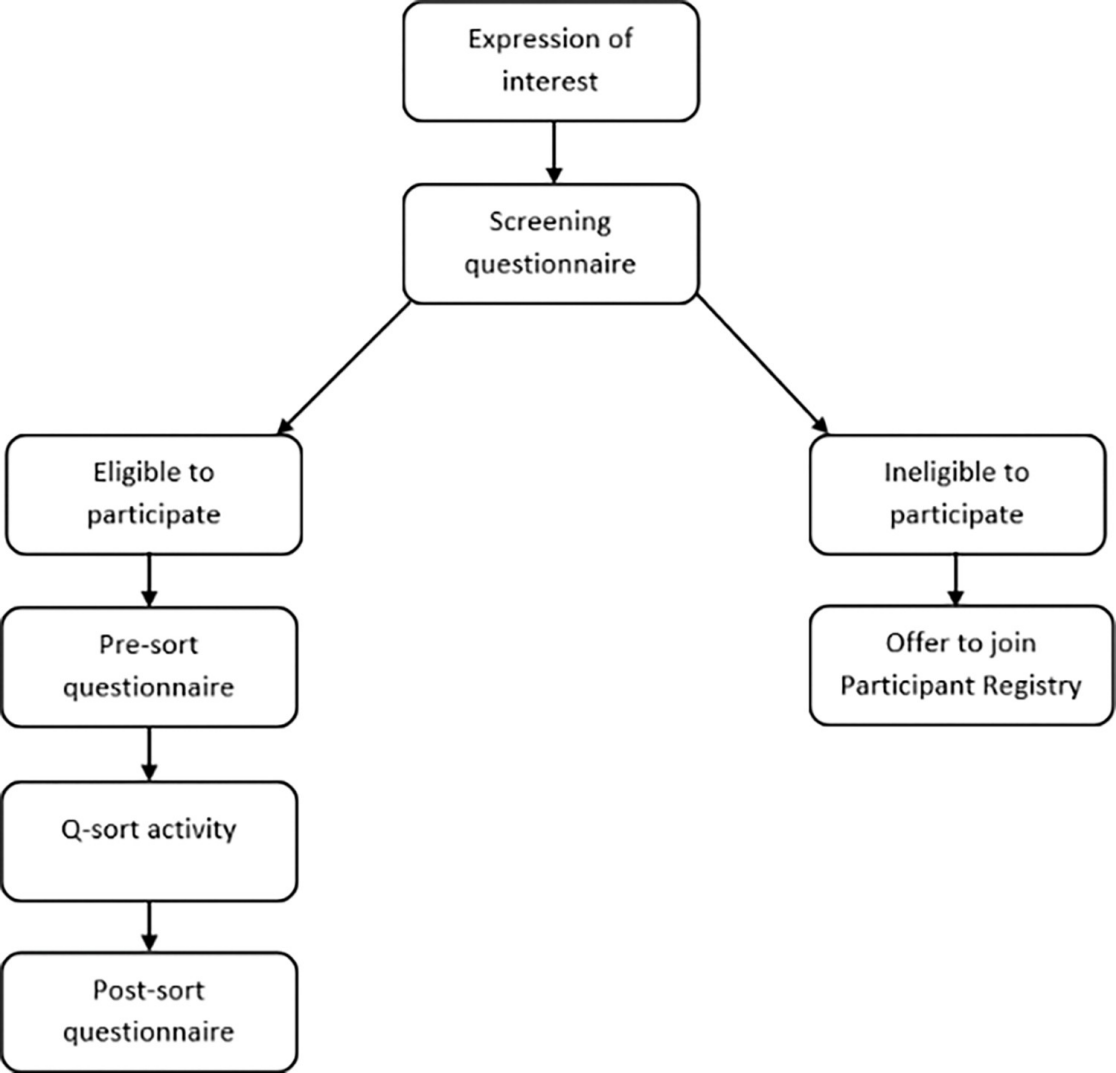
Flow diagram of the participant recruitment process.

The recruitment process for the proposed study is shown in Fig 2. In keeping with Q guidelines [27,28], the target sample size is 48 participants (12 individuals per group). Participants will be selected strategically using purposive and snowball sampling to capture a spectrum of perspectives about autistic burnout [22]. Recruitment for Q studies generally prioritises the selection of participants with diverse viewpoints over those with diverse demographic characteristics [28].

Research suggests that gender and autism awareness may be risk factors for autistic burnout [3]; therefore, individuals of all genders, with and without knowledge of autism and autistic burnout, will be invited to take part in the proposed study. Expressions of interest will be sought directly by advertising to relevant groups and organisations via social media, and indirectly through word of mouth. Recruitment will continue until target participant group numbers are achieved, or a broad range of perspectives about autistic burnout has been achieved.

#### Recruitment procedure

*Consent*. The Participant Information and Consent Form will be emailed to individuals who request further information about the study. The form includes the research aims and procedures, potential risks of participating, data storage and management policies, and how the findings will be disseminated. Prospective participants must sign and return the consent form before commencing and may withdraw from the study by signing and returning the Withdrawal of Consent section up to four weeks after their data have been collected.

### Materials and data collection

An Advisory Group of four, late-diagnosed autistic adults (3 female:1 male) with lived experience of autistic burnout was formed to provide advice about the study, share their insights on autistic burnout, and review the Q-set. All data collection methods are web-based and will not require participants to download or install software. The questionnaire data will be collected and managed using REDCap (Version 12.5.5) electronic data capture tools hosted at La Trobe University. QMethod Software (2022) [29] will host the online Q-sort, and interviews will be conducted via Zoom video conferencing. Participants may elect to use both the video and audio features, or audio only. Otter.ai software will be used to generate transcripts from the Zoom interviews that will be saved in separate Word documents for each participant. The first author may take notes during the interviews, and data will be stored in a password protected folder on her computer.

#### Concourse and Q-set

The concourse contains 267 items about autistic burnout sampled from a broad range of formal and informal sources including academic literature, social media posts and comments, blogs, YouTube, infographics, conference presentations, webinars, and casual conversations. From the concourse, a 71-item Q-set (S1 Table) encompassing three main themes was selected to represent the range of viewpoints about autistic burnout [22]:

1. Characteristics of autistic burnout (25 items).
2. How aspects associated with being autistic influence autistic burnout (‘internal factors’; 21 items).
3. How broader social and environmental factors influence autistic burnout (‘external factors’; 25 items).

The Q-set is impartial and contains a mix of positive and negative statements because sorting requires “…participants to impose their own meanings onto the items … and to infuse them with personal, or psychological, significance” [22, p64]. As the proposed study aims to examine common understandings among autistic and non-autistic individuals regarding burnout, general phrasing has been used (e.g., “Autistic burnout is a chronic condition”) rather than self-referential phrasing (e.g., “I feel exhausted during autistic burnout”) [22].

#### Pilot study

The study has been trialled with a six-member pilot group recruited from the researchers’ networks that represented all participant categories and included an autistic autism researcher. The group reviewed the Q-set and provided written feedback about the statements’ clarity, readability, and relevance. The group also tested a prototype of the online sorting activity and offered suggestions for improvement (S2 Table). Where possible, modifications were made in response to the group’s feedback. Post-sort interviews were not conducted with the pilot group participants.

#### Screening questionnaire

To ensure eligibility criteria are met, and representation of different opinions about autistic burnout, prospective participants will complete a screening questionnaire (S3 Table). All respondents will be asked to provide their name, age, and which group they represent. While participants can belong to multiple groups (e.g., a healthcare provider with an autistic friend), they will be required to choose the one that best describes them and will be directed to the relevant screening questionnaire for that group. All versions of the screening questionnaire will ask participants whether they know about autistic burnout and to rate their current knowledge on a Likert scale (Poor; Fair; Good; Very Good).

Screening questions will then be tailored for each group. For example, the autistic adult group will be asked if they have a formal autism diagnosis, age at diagnosis, and to indicate whether they have experienced autistic burnout. The healthcare professional group will be asked to specify their job title, level of experience working with autistic clients or patients, and approximately how many autistic people they have worked with. The parent and significant others group will be asked to indicate their relationship to the autistic adult and whether the autistic adult has experienced autistic burnout. The non-autistic adult group will be asked whether they self-identify as autistic and if they have ever experienced burnout (e.g., workplace; parenting).

Prospective participants will be ineligible if their chosen participant group has been filled, if their viewpoint has already been represented, or they do not meet the inclusion criteria for that group (e.g., their autistic child is under 18 years, or they do not have a formal autism diagnosis). Ineligible respondents will be thanked and invited to join the Olga Tennison Autism Research Centre Participant Registry to learn about other autism studies they may like to participate in. Eligible participants who are recruited into the study will be assigned a unique code to identify and collate their responses. Eligibility will be verified by checking the participants’ responses on the pre-sort questionnaire.

#### Pre-sort questionnaire

A pre-sort questionnaire (S4 Table) will be administered to explore underlying factors that may directly or indirectly influence participants’ viewpoints about the Q-set items. In addition to demographic information (age, gender), participants’ self-described attitudes toward neurodiversity will be collected [30] and additional questions customised for each group. For example, autistic adults will be asked if they have any co-occurring health conditions and to self-describe their experiences with healthcare professionals. Responses to the pre-sort questionnaire will facilitate factor interpretation during data analysis [22].

#### Q sort activity

Participants may complete either an online or hard copy Q sort. A study kit will be mailed to participants who request the hard-copy format. Watts and Stenner [22] have recommended using generous dimensions and plain-coloured materials to ensure they are easy to read and handle. The study kit will contain:

- Printed instructions (A4 size).
- A Q sorting grid (510mm x 760mm; 200gsm board; white).
- The 71-item Q set (randomly numbered; 70mm x 50mm each; 200gsm board; white).
- A blank Q grid (A4 size) for recording the final sorting configuration.

**Fig 3:**
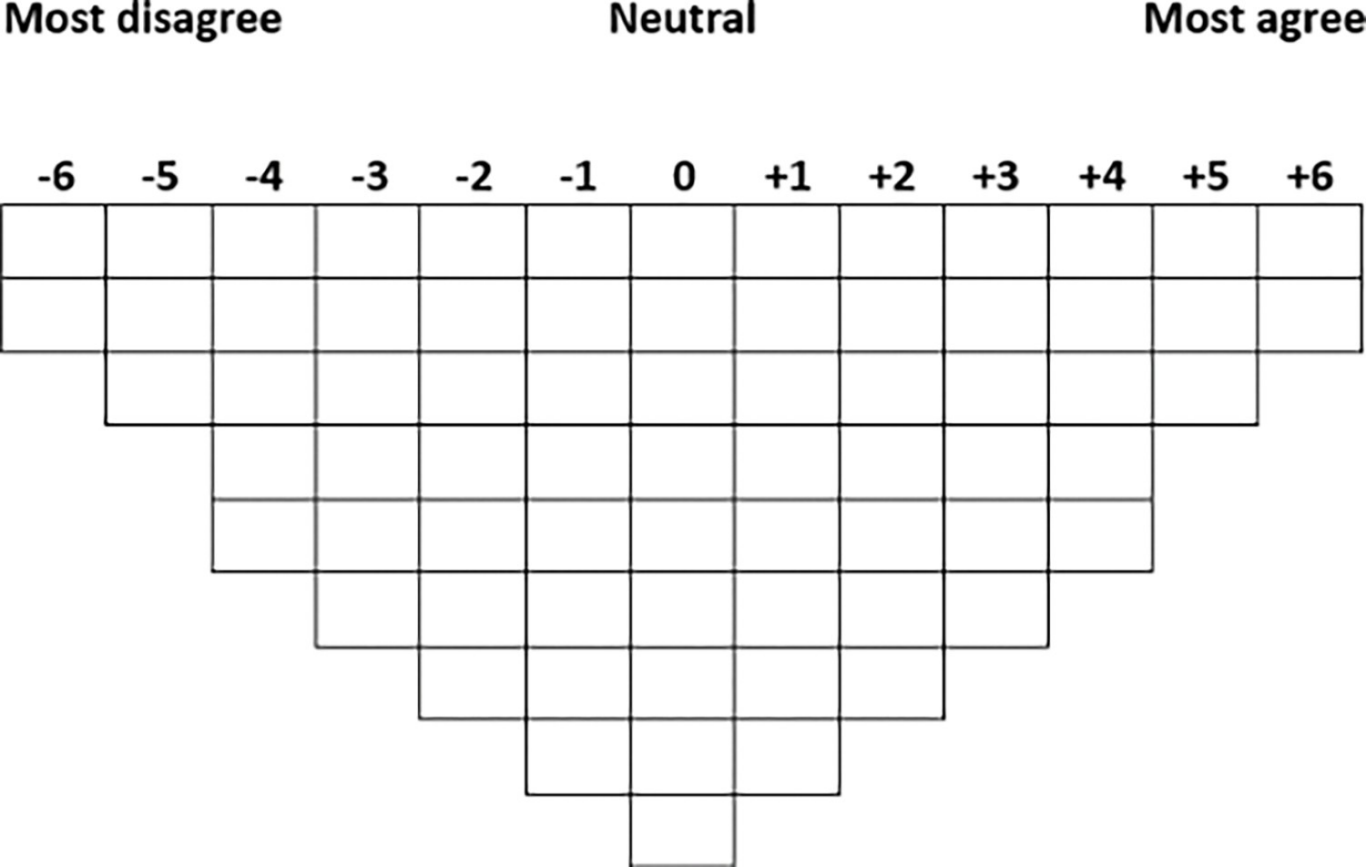
Example of the Q sorting grid for the proposed study.

The sorting procedure will be the same for the online and hard-copy formats. As the study is focused on exploring subjective understandings of autistic burnout, the condition of instruction will direct participants to include “I think” before each statement (e.g., *I think* autistic burnout is a chronic condition) [22]. First, participants will be asked to divide the statements among three provisional categories: ‘Agree’, ‘Disagree’, and ‘Neutral’. Sorting involves ranking the statements relative to each other onto a grid that resembles an inverted normal distribution (Fig 3). The statements are rated in columns ranging from Most Disagree (-) to Most Agree (+), with a midpoint (0) representing items about which the participants feel Neutral or unsure. A forced-choice (fixed) design will be used, meaning only one item can be allocated to each grid position. This design will require participants to place statements that elicit stronger responses towards the extremes of the grid. The order of items within each column is inconsequential [22].

Participants will sort the set of ‘Agree’ cards first, meaning they will rank the two statements they agree with *most* strongly in column +6, the three items they agree with slightly less strongly in column +5, and so on until all cards have been allocated. They will repeat the process with the ‘Disagree’ cards, except the two items they disagree with *most* strongly will be allocated to column -6, etc. The cards in the ‘Neutral’ pile will be sorted last. Participants can rearrange the statements until they are satisfied the Q sort best represents their point of view. Participants who complete the activity online will press ‘Submit’ to share their responses, while participants who complete the hard copy will email a digital photograph of their final configuration to the first author. Based on the pilot study, it is estimated that the activity will take approximately 15–30 minutes to complete, but participants will not have a time limit. All records will be stored securely.

#### Post-sort interview

The first author will contact each participant to arrange a ‘post-sort’ interview at a mutually beneficial date and time. According to Brown [31], the Q sort is “the skeleton of the subject’s attitude…[and] a conversation piece” [p200] that, through an interview, can reveal nuances and underlying motivations about people’s sorting choices. A short list of pre-prepared questions will be used to guide the interviews (e.g., ‘Thinking about the two statements you most agreed with, why were these statements particularly significant for you’?). The semi-structured interviews will be conducted via Zoom, recorded (with permission), and transcribed verbatim using Otter.ai software for subsequent analysis. Participants who prefer not to use Zoom may complete an online post-sort questionnaire instead via REDCap (S5 Table).

The post-sort interviews or questionnaire will first explore why the statements each participant placed at the extreme ends of the Q-grid (-6, +6) resonated most strongly with them. Card placement(s) that seem unusual relative to their general pattern of opinion [22] will also be examined. The post-sort interview is an opportunity to ask participants whether any aspects of autistic burnout were overlooked or missing from the Q-set. Participants who offer suggestions will be asked to indicate where on the grid they would have placed their statement, had it been included. This process is an essential element of a Q study that can reveal additional viewpoints about a topic [22]. Finally, participants will be asked to describe autistic burnout in their own words and to share any other comments or feedback about the study. It is estimated that each interview will take approximately 45 minutes. Follow-up interviews will not be conducted, and transcripts will not be shared with participants for feedback. In appreciation of their time and contribution, participants who complete all activities will be emailed a gift voucher to the value of $25 AUD (Australian participants) or $15 USD (international participants).

#### Data analysis

The quantitative data will be analysed using PQMethod software (Version 2.35) [32]. To capture the unique perspectives within each participant group, each group’s Q-sorts will first be analysed separately. Centroid factor analysis will be used to extract the factors because this technique considers all possible factor solutions rather than just the best mathematical option(s). Varimax rotation will be used to maximise the variance explained by the factor solution, and when deciding how many factors to retain, we will examine those with eigenvalues greater than 1.00 and at least two significant Q-sort loadings. The factor arrays will be flagged manually [22], and the resultant four sets of factors will represent the perspectives of participants *within* each group. Next, second-order factor analysis will be conducted to generate a set of ‘super factors’ that will highlight similarities and differences *between* the four groups. A two-tier analysis was chosen to ensure that no particular group’s perspective is privileged and to facilitate comparisons about each group’s perspectives, and the viewpoints overall.

The qualitative data collected with the pre-sort questionnaires and post-sort interviews will assist during factor interpretation [22], but themes will not be generated or analysed for the proposed study. The first author will conduct the initial factor interpretation which will be discussed among all members of the research team until general agreement is reached. The factor interpretations will be emailed to the study participants for review and an opportunity to provide further clarity or feedback, which may result in some final modifications to the interpretations.

## Discussion

Although autistic burnout can have significant negative consequences for the mental health and wellbeing of autistic people, there is little awareness about the condition outside the autistic community. This lack of knowledge can contribute to misdiagnosis, inadequate or inappropriate support, and loss of social and professional opportunities for autistic people. Early research has primarily focused on the lived experiences of autistic adults [1,2,4]. To the best of the authors’ knowledge, the current study is the first to holistically examine how autistic and non-autistic adults understand autistic burnout and will highlight potential knowledge disparities that can be targeted for action. The study is designed to characterise all aspects of autistic burnout to improve recognition of symptoms and identify risk and protective factors. It is anticipated that the findings will yield positive outcomes for various stakeholder groups.

### Autistic adults

Anecdotal accounts of autistic burnout have been shared informally for approximately two decades, but research is still in its infancy. The findings will further validate autistic people’s lived experiences and improve awareness among autistic people who may not already know about autistic burnout. Insights from this study could inform strategies to help autistic people recognise the signs of burnout in themselves and their autistic peers.

### Parents, significant others, and non-autistic adults

The findings from this study will help families and friends of autistic people identify when their autistic loved one shows signs of impending burnout (e.g., greater incidence of meltdowns or shutdowns, extreme fatigue) [2,4] and offer appropriate support. Relatives of autistic people have previously described feeling guilt and regret about not recognising and understanding the challenges faced by their autistic loved ones, thus failing to offer appropriate support [33]. Among non-autistic adults, the study findings may highlight the impact of negative autism stereotypes, stigma, and discrimination of autistic individuals and the importance of acceptance and understanding.

### Healthcare professionals

Healthcare professionals can play a crucial role in detecting and treating autistic burnout. The findings may inform the development of a screening tool to assist healthcare professionals in recognising the symptoms of autistic burnout in their clients. The findings may provide insight into what questions to ask relevant to burnout and how to best support their autistic clients, contributing to improved patient care and professional skill development.

### Research community

The study will contribute to the fulfilment of a Doctor of Philosophy. Its findings will be presented at relevant stakeholder and research conferences and published in peer-reviewed journals and conference abstracts associated with presentations. Insights from this research will enhance the current definitions and understanding of autistic burnout and inform future awareness and education initiatives. The findings may inform the development of a valid and reliable measure of autistic burnout and offer new hypotheses for testing in future research.

## Supporting information

S1 Table List of statements to be sorted by participants. (DOCX)

S2 Table Summary of feedback by pilot study participants. (DOCX)

S3 Table Screening questionnaire. (DOCX)

S4 Table Pre-sort questionnaire. (DOCX)

S5 Table Post-sort interview guide/online questionnaire. (DOCX)
