# Supplementary material for: Examining subjective understandings of autistic burnout using Q methodology: A study protocol
Source: PLoS One. 2023 May 19;18(5):e0285578. doi: 10.1371/journal.pone.0285578 (PMC10198508; doi:10.1371/journal.pone.0285578)
Supplement: S1 Table — (DOCX) [file pone.0285578.s001.docx]

**S1. Table A. List of Statements to be Sorted by Participants.**

| Item | Q-set Statement |
| --- | --- |
| 1 | I can clearly describe what autistic burnout is |
| 2 | It's possible to recover from autistic burnout by taking a short break (e.g., a long weekend or a few days off) |
| 3 | A person experiencing autistic burnout can still look after themselves (e.g., bathe, cook, eat) |
| 4 | An early autism diagnosis can reduce the likelihood of developing autistic burnout |
| 5 | Autism acceptance will improve in the future |
| 6 | Autism can be self-diagnosed |
| 7 | Autistic burnout is the same thing as autistic regression |
| 8 | There is a difference between autistic burnout and depression |
| 9 | Autistic burnout can be self-diagnosed |
| 10 | Autistic burnout can lead to suicidal thoughts and/or behaviours |
| 11 | Autistic burnout can occur at any age (i.e: childhood, adolescence, adulthood, late adulthood) |
| 12 | Autistic burnout can prevent autistic people from reaching their potential |
| 13 | Autistic burnout is a neurological condition |
| 14 | Autistic burnout is just a normal part of being an autistic person |
| 15 | Autistic burnout is often misdiagnosed or mistaken for other conditions (e.g. depression, anxiety, chronic fatigue syndrome) |
| 16 | Autistic burnout is caused by trauma (e.g., victimisation, bullying) |
| 17 | Autistic burnout is the same as any other types of burnout (e.g., workplace; parenting) |
| 18 | It can't be autistic burnout unless the person experiences symptoms for at least 3 months |
| 19 | Autistic females are more likely to experience autistic burnout than autistic males |
| 20 | Autistic people only act as if they're burned out to get attention |
| 21 | Autistic people need to work twice as hard as non-autistic people to prove they're equally competent |
| 22 | Being highly empathetic makes a person more vulnerable to developing autistic burnout |
| 23 | Autistic people with high support needs can't develop autistic burnout |
| 24 | Autistic people with lower support needs can't develop autistic burnout |
| 25 | Difficulties describing emotions and feelings can contribute to autistic burnout |
| 26 | Difficulties noticing hunger, pain or tiredness in your body can lead to autistic burnout |
| 27 | During autistic burnout, autistic people can still communicate online, even if they can't do so in person |
| 28 | Spending too much time on special interests can contribute to autistic burnout |
| 29 | Feeling ashamed of being autistic contributes to autistic burnout |
| 30 | In general, healthcare professionals know a lot about autistic people |
| 31 | Healthcare professionals receive enough training about autism |
| 32 | Healthcare professionals respect autistic people's self-expertise and knowledge |
| 33 | Healthcare providers have preconceived or stereotypical ideas about autistic people |
| 34 | I know how to take care of myself or someone who is experiencing autistic burnout |
| 35 | I'm confident I could recognise the symptoms of autistic burnout (in myself or others) |
| 36 | It's possible for a person to regain their pre-burnout levels of functioning (cognitive, physical) |
| 37 | It's possible for autistic people to participate in their special interests during a period of burnout |
| 38 | Recovery from autistic burnout is a case of mind over matter |
| 39 | Social avoidance is a positive way of preventing autistic burnout |
| 40 | Sometimes people who try to help autistic people do more harm than good |
| 41 | Some treatments can make autistic burnout worse (e.g., cognitive behavioural therapy [CBT]; antidepressants) |
| 42 | The autistic community are the best source of support during autistic burnout |
| 43 | There are only negative consequences of autistic burnout |
| 44 | There is a spectrum of autism burnout severity (e.g., mild-moderate-severe) |
| 45 | There is enough awareness about autism |
| 46 | It's a person's own fault if they burn out |
| 47 | Others make autistic people feel guilty for asking for accommodations or rest |
| 48 | The pandemic has helped reduce the risk of developing autistic burnout |
| 49 | Sensory overload is a main risk factor for autistic burnout |
| 50 | "Taking the mask off" just leads to autistic burnout in other ways |
| 51 | The effort of masking/hiding autistic traits is the main risk factor for autistic burnout |
| 52 | Autistic people are capable of seeking medical/health care during periods of burnout |
| 53 | Autistic burnout and fatigue are the same thing |
| 54 | It's possible to reach autistic burnout suddenly, without much warning |
| 55 | A person's personality influences how vulnerable they are to developing autistic burnout |
| 56 | All autistic people know what autistic burnout is |
| 57 | Autistic burnout is a type of mental illness |
| 58 | Autistic burnout could be prevented if there was more acceptance of autism in society |
| 59 | The world isn't designed for autistic people to thrive |
| 60 | It's common for people to experience autistic burnout only once |
| 61 | Autistic people who don't experience burnout are alienated by the autistic community |
| 62 | Sleep is more difficult during burnout |
| 63 | Autistic people don't seek healthcare during burnout because they're afraid of being misdiagnosed |
| 64 | Autistic burnout affects all areas of a person's life |
| 65 | During autistic burnout, it's common for a person to lose their ability to speak |
| 66 | Autistic people burn out because they put the needs of others before their own |
| 67 | Healthcare professionals know what to do when autistic people are in crisis |
| 68 | Autistic people hide being in burnout so they don't lose their friends or job |
| 69 | Autistic burnout can't be valid until it is an official medical diagnosis |
| 70 | Being able to use self-regulation strategies like stimming can protect against burnout |
| 71 | The autistic community are the experts about autistic burnout |
