# Supplementary material for: Examining subjective understandings of autistic burnout using Q methodology: A study protocol
Source: PLoS One. 2023 May 19;18(5):e0285578. doi: 10.1371/journal.pone.0285578 (PMC10198508; doi:10.1371/journal.pone.0285578)
Supplement: S2 Table — (DOCX) [file pone.0285578.s002.docx]

**S2. Table B. Summary of Feedback by Pilot Study Participants**

| **Participant Comments** | |
| --- | --- |
| 1 | The instructions for the activity were clear, straightforward, and contained sufficient detail to complete the task. |
| 2 | Most participants found the online sorting activity fun, interactive, and engaging. |
| 3 | The forced choice design was challenging for some participants, but ultimately enabled them to evaluate their opinions more closely. |
| 4 | The visible timer made some participants feel stressed. |
|  |  |
| **Recommendations** | |
| 1 | Rearrange some paragraphs so the instructions flow better. |
| 2 | Do not include a timer for the activity. |
| 3 | Include an alternative to the online activity, if possible. |
| 4 | Minor formatting changes to headings and font size. |
| 5 | Clearly indicate that the order of the cards within each numbered column is not important. |
