# Supplementary material for: Examining subjective understandings of autistic burnout using Q methodology: A study protocol
Source: PLoS One. 2023 May 19;18(5):e0285578. doi: 10.1371/journal.pone.0285578 (PMC10198508; doi:10.1371/journal.pone.0285578)
Supplement: S3 Table — (DOCX) [file pone.0285578.s003.docx]

**S3. Table C. Screening Questionnaire.**

| Please answer the following questions: | |
| --- | --- |
| **Which group describes you?** | |
| 1 | I am an autistic adult. |
| 2 | I am a non-autistic adult. |
| 3 | I am a healthcare professional. |
| 4 | I am the parent, partner, friend or family member of an autistic adult. |
|  | *(Participants will be directed to the appropriate group)* |
|  |  |
| **Autistic adults’ group** | |
| 1 | What is your name? |
| 2 | How old are you? |
| 3 | Do you have a formal diagnosis of autism? |
| 4 | Do you know about autistic burnout? |
| 5 | How would you rate your knowledge?  Poor, Fair, Good, Very Good |
| 6 | Have you ever experienced autistic burnout? |
| 7 | Can you participate in this study during xxxx? (You will be able to choose which day suits you best). |
|  |  |
| **Non-autistic adults’ group** | |
| 1 | What is your name? |
| 2 | How old are you? |
| 3 | Do you have a formal diagnosis of autism or self-identify as autistic? |
| 4 | Have you ever experienced any form of burnout? (for example, job-related, parenting, student). |
| 5 | *(If yes)* Please specify which type*.* |
| 6 | Do you know about autistic burnout? |
| 7 | How would you rate your knowledge?  Poor, Fair, Good, Very Good |
| 8 | Can you participate in this study during xxxx? (You will be able to choose which day suits you best). |
|  |  |
| **Healthcare professionals’ group** | |
| 1 | What is your name? |
| 2 | Are you a healthcare professional? (e.g., GP, psychologist, psychiatrist, nurse, speech pathologist, occupational therapist). |
| 3 | Please specify which type. |
| 4 | Have you had any experience working with autistic clients or patients? |
| 5 | Please describe your level of experience (e.g., weeks, months, years). |
| 6 | Approximately how many autistic clients / patients have you worked with? |
| 7 | Do you know about autistic burnout? |
| 8 | How would you rate your knowledge?  Poor, Fair, Good, Very Good |
| 9 | Can you participate in this study during xxxx? (You will be able to choose which day suits you best). |
|  |  |
| **Parent and significant others’ group** | |
| 1 | What is your name? |
| 2 | How old are you? |
| 3 | Are you the parent, partner, friend or relative of an autistic adult (i.e., who is 18 years or older)? |
| 4 | Please specify your relationship to this autistic adult. |
| 5 | Do you know about autistic burnout? |
| 6 | How would you rate your knowledge?  Poor, Fair, Good, Very Good |
| 7 | Has the autistic adult in your life ever experienced autistic burnout?  Yes, No, I don’t know |
| 8 | Can you participate in this study during xxxx? (You will be able to choose which day suits you best). |
