# Supplementary material for: Examining subjective understandings of autistic burnout using Q methodology: A study protocol
Source: PLoS One. 2023 May 19;18(5):e0285578. doi: 10.1371/journal.pone.0285578 (PMC10198508; doi:10.1371/journal.pone.0285578)
Supplement: S4 Table — (DOCX) [file pone.0285578.s004.docx]

**S4. Table D. Pre-sort Questionnaire.**

| Please answer the following questions: | |
| --- | --- |
| **Demographic information** | |
| 1 | What is your name? |
| 2 | Which best describes your gender?  Male, Female, Non-binary, I prefer to self-describe |
| 3 | Which of the following describes you?  I am an autistic adult.  I am the parent or caregiver of an autistic adult.  I am the partner, friend, sibling, or other family member of an autistic adult.  I am a healthcare professional.  I am a non-autistic adult.  *(Participants will be directed to the appropriate group)* |
|  |  |
| **Autistic adults’ group** | |
| 1 | How old were you when you received your autism diagnosis? |
| 2 | Do you have any co-occurring mental or physical health conditions? |
| 3 | *(If yes)* Please specify which type*.* |
| 4 | Have you experienced autistic burnout? |
| 5 | *(If yes)* How old were you? |
| 6 | Have you experienced autistic burnout more than once? |
| 7 | *(If yes)* How many times? |
| 8 | How long did the episode(s) last? |
| 9 | Did autistic burnout lead to your autism diagnosis? |
| 10 | How would you describe your experiences with healthcare professionals? |
| 11 | How would you describe your knowledge about autism? |
| 12 | Would you describe autism as a disorder that should be treated so it becomes less obvious, or as a natural neurological difference that should be accepted? |
|  |  |
| **Parent / caregivers’ group** | |
| 1 | How many children do you have? |
| 2 | How many of your children are autistic? |
| 3 | How old was your child/ren when they were diagnosed? |
| 4 | Does/do your child/ren have any co-occurring mental or physical health conditions? |
| 5 | *(If yes)* Please specify. |
| 6 | Has your child experienced autistic burnout?  Yes, No, I don’t know. |
| 7 | *(If yes)* At what age did their burnout occur? |
| 8 | Has your child experienced autistic burnout more than once? |
| 9 | *(If yes)* How many episodes? |
| 10 | How long did your child’s burnout last? |
| 11 | As their parent / caregiver, how would you describe your autistic child’s experiences with healthcare professionals? |
| 12 | How would you describe your knowledge about autism? |
| 13 | Would you describe autism as a disorder that should be treated so it becomes less obvious, or as a natural neurological difference that should be accepted? |
|  |  |
| **Partners’ or other relationship group** | |
| 1 | What is your relationship to this autistic person? |
| 2 | Does your loved one have any co-occurring mental or physical health conditions? |
| 3 | *(If yes)* Please specify which type*.* |
| 6 | Has your child experienced autistic burnout?  Yes, No, I don’t know. |
| 7 | *(If yes)* At what age did their burnout occur? |
| 8 | Have they experienced autistic burnout more than once? |
| 9 | *(If yes)* How many episodes? |
| 10 | How long did their burnout last? |
| 11 | From your knowledge, how would you describe your autistic child’s experiences with healthcare professionals? |
| 12 | How would you describe your knowledge about autism? |
| 13 | Would you describe autism as a disorder that should be treated so it becomes less obvious, or as a natural neurological difference that should be accepted? |
|  |  |
| **Healthcare professionals’ group** | |
| 1 | What type of health profession are you in? (e.g., GP, psychologist, psychiatrist, nurse, speech pathologist, occupational therapist). |
| 2 | Do you have ANY experience working with autistic clients / patients? |
| 3 | How many years have you worked with autistic clients / patients? |
| 4 | Approximately how many autistic clients / patients have you worked with? |
| 5 | Do you feel that you have enough training and knowledge about autism? (Please describe). |
| 6 | Would you describe autism as a disorder that should be treated so it becomes less obvious, or as a natural neurological difference that should be accepted? |
|  |  |
| **Non-autistic adults** | |
| 1 | Have you ever experienced burnout? (e.g., from work, parenting, study) |
| 2 | (If yes) At what age did burnout occur? |
| 3 | How long did the period of burnout last? |
| 4 | Have you experienced burnout more than once? |
| 5 | (If yes) How many times? |
| 6 | Do you have any mental or physical health conditions? |
| 7 | *(If yes)* Please specify which type*.* |
| 8 | How would you describe your experiences with healthcare professionals? |
| 9 | How would you describe your knowledge about autism? |
| 10 | Would you describe autism as a disorder that should be treated so it becomes less obvious, or as a natural neurological difference that should be accepted? |
