# Supplementary material for: Examining subjective understandings of autistic burnout using Q methodology: A study protocol
Source: PLoS One. 2023 May 19;18(5):e0285578. doi: 10.1371/journal.pone.0285578 (PMC10198508; doi:10.1371/journal.pone.0285578)
Supplement: S5 Table — (DOCX) [file pone.0285578.s005.docx]

**S5. Table E. Post-sort Interview Guide / Online Questionnaire.**

| We hope you enjoyed completing the Q-sort activity!  In this final task we’d like to explore why certain statements resonated strongly with you.  Your responses will help us to better understand and analyse your Q-sort. Please include as much detail as possible. | |
| --- | --- |
| 1 | Here is a reminder of the statements you agreed with most strongly *(show participant their card placements for columns +6 and +5).* |
| 2 | First, looking at the column on the right, can you please explain why you agreed most strongly with these 2 items? In your opinion, why were these statements especially significant? |
| 3 | You also agreed very strongly with the 3 statements in the left column. Can you please explain why you had strong opinions about them? |
| 4 | Below is a reminder of the statements you disagreed with most strongly *(show participant their card placements for columns -6 and -5).* |
| 5 | Looking first at the 2 statements on the left, could you please explain why you disagreed most strongly with these items? In your opinion, why were they especially significant? |
| 6 | You also disagreed with the 3 statements in the other column quite strongly. Can you please explain your opinions about those items in more detail? |
| 7 | In the Q-sort, we tried to include a range of different perspectives about autistic burnout. Were there any items you feel we missed and should have included? |
| 8 | *(If yes)* If the item(s) had been included, in which numbered column on the Q-sort grid would you have placed it (them)? |
| 9 | Could you please describe your overall understanding of autistic burnout? What does autistic burnout mean, in your opinion? |
| 10 | Please use this space to share anything else you’d like us to know about autistic burnout or the study. |
|  |  |
| Thank you for taking the time to participate in our study! | |
